# Depression, Strokes and Dementia: New Biological Insights into an Unfortunate Pathway

**DOI:** 10.1155/2011/649629

**Published:** 2011-12-15

**Authors:** Antoine M. Hakim

**Affiliations:** ^1^Neuroscience Research, Ottawa Hospital Research Institute, Ottawa, ON, Canada K1Y 4E9; ^2^Canadian Stroke Network and Center for Stroke Recovery, Ottawa, ON, Canada K1G 5Z3; ^3^Division of Neurology, University of Ottawa, 2413-451 Smyth Road, Ottawa, ON, Canada K1H 8M5

## Abstract

The literature emphasizes the risk of depression after a stroke. Less well known is the fact that depression may be as big a risk factor for strokes as hypertension, particularly in the older age group. This article reviews the risk for stroke and cognitive impairment consequent to depression, and describes the cardiovascular and immunological mechanisms that would appear to link depression to its cerebrovascular consequences. As well, the article refers to the brain imaging signatures that may allow prediction of impending brain injury. Finally, some questions that might be explored by future research are suggested, and some practical means to identify and help those at risk for the development of depression-associated vascular disease of the brain are suggested.

## 1. Introduction

It is now reliably proven that strokes lead to both depression and diminished cognitive ability. Perhaps less well known is the fact that depression is a major risk factor for stroke. This paper is a selective review of the rapidly expanding literature linking emotional states to vascular health through their influence on the immune system. It is the result of multiple literature searches through PubMed and other search engines spanning the period from 2000 to 2011. Preference was given to meta-analyses and large health surveys. The articles included in this paper were selected because they contributed to understanding the mechanisms by which depression may lead to cognitive impairment through the intermediate step of vascular damage to the brain and provided new insights into the mechanisms by which this process may occur. The paper identifies questions that remain unanswered and suggests some practical approaches that may help to break the link suggested in the title.

## 2. Strokes and Depression

Emerging depression is a common problem after stroke [1]. The best estimates are that approximately one-third of people who have had a stroke will display poststroke depression [2], which in addition to being characterized by low mood, alterations in weight, appetite and sleep pattern, is associated with reduced perception of quality of life and can lead to suicidal ideation [3, 4].

There would seem to be some gender differences in the likelihood of post-stroke depression. Thus, while depression at baseline in men and women is found to be similar, women appear more likely to report depression after stroke even when adjusting for age, stroke severity, and comorbidities [5]. It is also a matter of debate whether the likelihood of poststroke depression is determined by premorbid psychosocial factors, lesion size and location, biologic mechanisms such as disruption of neurotransmission and release of cytokines, or a combination of these factors [6].

## 3. Strokes and Cognitive Decline

It is now well established that cognitive impairment can be a consequence of brain infarcts. The risk of not attaining healthy aging, adjusted for age, increased twofold in participants with brain infarcts [7]. In the Nun study [8], the presence of a single lacune at autopsy significantly increased the probability of dementia. Imaging and other studies have demonstrated that lesions in the white matter are common, related to hypertension, increase the risk of mild cognitive impairment (MCI), and more than double the risk of dementia [9–12]. The Framingham offspring study showed that, depending on how extensive the white matter hyperintensities (WMHs) were, the hazard ratio for dementia varied between 2.22 and 6.12, independently of vascular risk factors [13]. Nor is cognitive decline limited to small vessel disease (SVD) and lacunar strokes: 50% of those who suffer large strokes exhibit cognitive deficits within months [14]. Thus, the assertion that strokes lead to cognitive decline is true for both large strokes and smaller white matter lesions.

## 4. Depression and Cognitive Decline

In a recent systematic review of the factors associated with cognitive decline in later life, Plassman and her colleagues reviewed all large observational studies and randomized controlled trials published over 25 years ending in 2009 [15]. They concluded that there was indeed an association between depression and cognitive decline. Köhler and colleagues reported that half of the patients with major depressive disorder exhibited generalized cognitive impairment [16]. This pattern and course of cognitive impairment in late-life depression confirmed what Dufouil and colleagues had previously reported: a strong cross-sectional association between depressive symptomatology and poor cognitive performance [17, 18]. In 2006, Ganguli and colleagues had also concluded their 12-year prospective epidemiological study by stating that depressive symptoms were associated with cognitive impairment [19]. Thus, with rare exceptions [20], the consensus appears to indicate a strong negative influence of depression on cognitive function. It is important to keep in mind that the majority of the studies that found an association between depression and cognitive deficits determined the presence of depression through semistructured interviews rather than clinical evaluations. Thus, observations of apathy, anxiety, and sleep difficulties may have been labelled as depression. Still, the mechanisms that may underlie this association between depression and cognitive decline have recently been receiving a lot of attention.

## 5. Depression as a Risk Factor for Stroke

The traditional risk factors that lead to strokes are well known: advancing age, hypertension, smoking, hypercholesterolemia, obesity, and diabetes. Less well known is the association between depression and both strokes and dementia, which is seen in reports from both large cohorts and smaller observation studies. In a 2005 paper, Williams reviewed all the studies specifically linking depressive symptoms with increased risk of strokes and concluded that depression is a strong risk factor for stroke [21]. In a 6-year prospective cohort study of nearly 2500 older adults, Ostir and colleagues found that stroke risk increased with the severity of depression and, conversely, that positive affect scores had a strong inverse relationship with stroke [22]. Data from the National Health and Nutrition Examination Survey (NHANES) likewise showed that depression symptoms at baseline were associated with increased stroke risk even after adjusting for vascular risk factors [23], while a study by Krishnan and colleagues, using data from the Leukoaraiosis and Disability (LADIS) study, revealed that depression accounted for 12% of the variance in stroke incidence beyond the contribution of the vascular risk factors [24]. A Danish study showed that stroke risk in older patients with depression was increased by 22%, and the risk was not present in patients with bipolar disorder, making depression a unique mental state that leads to strokes [25]. Sarah Vermeer in her review of the factors associated with brain white matter hyperintensities reported that the incidence of covert strokes in patients with depression was 46%, while that in patients with hypertension was 43%, and in individuals with cerebrovascular risk factors it was 39% [26]. Although one must keep in mind that the presence of vascular involvement in organs other than brain may contribute to depression and strokes, these data suggest that the association between stroke and depression is at least as strong as that with more traditional risk factors.

The age at which the depression begins appears to influence the vulnerability to stroke. Herrmann and colleagues from Oxford University identified 30 eligible studies investigating white matter changes in late life depression and concluded from their meta-analysis that the odds of having white matter changes were over 4 for late-compared with early-onset depression [27]. There may also be a gender difference in the vulnerability to stroke in the depressed state. In the Women's Health Initiative, women with depression but no history of cardiovascular disease were not at increased risk of stroke, whereas those with a history of prior cardiovascular disease were at 45% increased risk of stroke [28]. Nonetheless, it is increasingly evident that depression is a strong risk factor for stroke, but this link may be modulated by both age and gender.

## 6. Mechanisms Linking Depression to Vascular Brain Disease and Cognitive Decline

**Figure 1:**
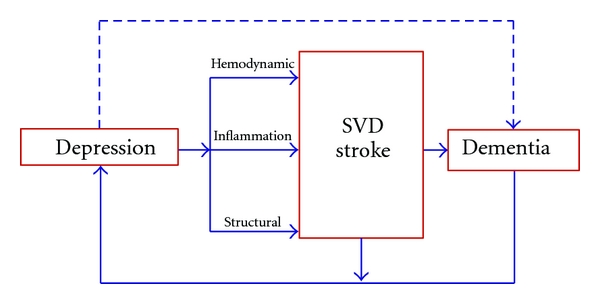
The pathway from depression to dementia and the mechanisms that play a role. Small Vessel disease (SVD) and stroke may or may not be the exclusive pathway from depression to dementia, as the upper line suggests.

There are intriguing suggestions in the literature as to the mechanisms that may underlie depression's role in leading to strokes and cognitive impairment. These are portrayed in (Figure 1).

Several authors have highlighted the vascular, metabolic, and inflammatory changes that accompany sadness and depression, and recent imaging studies have added to the weight of the evidence by suggesting that depression leads both to weakening of the subcortical white matter and to occlusion of small brain vessels. The following mechanisms may link depression to vascular disease.

### 6.1. Hemodynamic

The cardiovascular system responds to our state of mind. Prkachin and colleagues interviewing undergraduate students concluded that systolic and diastolic blood pressures as well as stroke volume differentiated among emotions [29]. In a study by Sinha and colleagues which examined the cardiovascular mechanisms governing differential blood pressure changes during the emotions of joy, sadness, fear, and anger, sadness resulted in a distinct pattern, with moderate increases in blood pressure and vascular resistance, and a decrease in cardiac output [30]. These changes correspond to what is known about the vulnerability of the brain to vascular risk factors: in a comparison of Interstroke and InterHeart data, it was shown that the brain is far more sensitive to rises in blood pressure than the heart [31]. Thus, sustained cardiovascular changes of the pattern reported by Sinha and colleagues may well contribute to the development of ischemic brain damage.

### 6.2. Inflammatory

Potentially the most relevant mechanism linking depression to both stroke and cognitive decline is the response of the immune system to sustained depression. Dantzer and coworkers reported that interleukin-6, a major proinflammatory cytokine, was increased in the blood of depressed patients [32], and a recent meta-analysis concluded that only the basal levels of IL-6 and tumour necrosis factor-alpha (TNF-*α*) were significantly raised in major depression [33]. These findings led Leonard to suggest that depression arises from a dysfunction of the immune system [34]. Zhu and colleagues have reported that proinflammatory cytokines can acutely regulate neuronal serotonin [35], thus linking inflammatory systems to the more traditional understanding of the neurochemistry of depression, a concept further expanded by Steiner and colleagues [36].

If depression is associated with proinflammatory cytokines, these same agents are now known to be associated with cognitive impairment. This link was recently reviewed by Gorelick [37]. Trollor and colleagues reported from the Sydney Memory and Aging Study that levels of TNF-*α* and serum amyloid A were higher in participants with MCI than in cognitively normal individuals [38], confirming earlier reports to that effect [39, 40]. Their studies however showed that this association was influenced by gender. TNF-*α* has also been shown to be higher in vascular dementia than in Alzheimer's disease [41]. Although the raised level of C-reactive protein (CRP) in individuals with dementia has been known for some time [42, 43], a recent study where patients with TIA or minor stroke were followed for 3 months showed no association between circulating inflammatory markers that included CRP, interleukin-1 receptor antagonist, and interleukin-6 but not TNF-*α* and recurrence of the vascular events [44].

The fact that cytokines are elevated in depression and cognitive impairment does not necessarily confer to them an etiologic role in those settings. However, once elevated in the setting of depression, TNF-*α*, by reducing endothelial nitric-oxide synthase (eNOS) expression, can lead to neuronal and vascular injury through a decrease in nitric oxide bioavailability and the formation of reactive oxygen species. As well, TNF-*α* changes protein expression and/or activity in macrophages and monocytes leading to increased cholesterol uptake and the generation of foam cells. Through these actions, TNF-*α* damages endothelial cells leading to reduced integrity of the endothelial barrier and increased fibrinolysis and apoptosis of these cells. As well, it affects vascular smooth muscle cells, leading to their proliferation with consequent reduction of the vascular caliber. Thus, TNF-*α* is a major driver of endothelial dysfunction and atherosclerosis through a variety of mechanisms that were recently reviewed [45]. Although the review by Kleinbongard and colleagues referred to here highlighted the role of TNF-*α* in determining the size of a myocardial infarct and subsequent remodelling, many of the pathways identified are equally relevant to brain ischemia. These pathological actions of TNF-*α* are aggravated by aging, smoking, and obesity, which are associated with a chronic low-grade increase in TNF-*α* [46], suggesting that a positive feedback loop may occur in some clinical settings that accentuate the vascular damaging effects of both TNF-*α* and the more traditional vascular risk factors.

### 6.3. Structural Changes

A recent imaging study has shown that depressed individuals show microstructural brain abnormalities in the regions known to be vulnerable to the development of WMH in the ischemic setting. Investigators, looking for microstructural changes in midbrain regions in subjects suffering from major depression, reported finding white matter changes in deep cortical white matter structures [47]. These affected regions were not in the investigators' a priori areas of evaluation, but the intensity of changes in them could not be ignored, making it likely that depression leads directly to structural changes in the white matter or at a minimum can render it more vulnerable to such damage. Interestingly, the deep cortical white matter is also reported to suffer microstructural abnormalities in normal aging [48], perhaps indicating why older individuals may be more vulnerable to the development of WMH in the setting of depression.

## 7. Questions for Future Research

Studies reviewed here show strong binary associations between depression and MCI, between depression and stroke, and between stroke and MCI. Importantly, the paper makes strong suggestions on the role the immune system may play in the pathway from depression to dementia. A study is needed that can prospectively determine whether the pathway from depression to MCI is exclusively through vascular brain damage and what immune biomarkers may be important players in this sequence. Another assumption that pervades the literature and could be tested in this study is that depression causes strokes predominantly through SVD and white matter rather than cortical injury. This paper also suggests that the age at which depression starts, its severity, and the gender of the individual affected are relevant to the subsequent development of cognitive impairment. A study such as the proposed here could not only measure the relative incidence of white matter versus cortical damage in the setting of depression and their association with dementia, but also address the roles of age, gender, and the traditional risk factors in determining the progression from depression to dementia.

New therapeutic insights would also arise from such a clinical study. For example, the concept that inflammation is associated with the depressed state of mind and can lead to vascular and structural injury to the brain has led to the suggestion that, in the treatment of depression, nonsteroidal anti-inflammatory medication in addition to antidepressants may be more effective than antidepressants alone [49, 50]. A more recent and very limited study in stroke patients reported significant improvement in stroke symptoms following the administration of a TNF-*α* inhibitor [51]. The study proposed here would not only allow measurement of inflammatory mediators, but may also allow testing whether the pathway from depression to MCI can be interrupted concomitant with a decrease in the appearance of WMH and cognitive decline.

## 8. Breaking the Cycle from Depression to Dementia

The onset of cognitive impairment, especially if associated with a stroke, would be expected to diminish the prospects for recovery and increase the burden of care. Antidepressants may enhance cognitive recovery following stroke, independently of their effect on depression [52]. Because of this, nursing homes and elder care facilities are attempting to detect depression in their residents and correcting it [53], so much so that excesses in antidepressant treatments have been identified [54]. Martha Daviglus and her colleagues in an NIH consensus statement reported that whereas the association of cognitive decline with living alone or being without a partner is inconsistent, “a robust association exists between the loss of a spouse and cognitive decline” [55]. This raises the possibility that individuals who have recently lost a spouse, provided that ethical boundaries are respected, may welcome involvement in a structured psychosocial program aimed at decreasing the likelihood of depression and increasing the chances of treatment. Depression in the setting of sudden bereavement is an understandable state of mind, but it does not diminish the fact that it may be harmful to brain function.
